# Exome sequencing identifies nonsegregating nonsense *ATM* and *PALB2* variants in familial pancreatic cancer

**DOI:** 10.1186/1479-7364-7-11

**Published:** 2013-04-05

**Authors:** Robert C Grant, Wigdan Al-Sukhni, Ayelet E Borgida, Spring Holter, Zaheer S Kanji, Treasa McPherson, Emily Whelan, Stefano Serra, Quang M Trinh, Vanya Peltekova, Lincoln D Stein, John D McPherson, Steven Gallinger

**Affiliations:** 1 Samuel Lunenfeld Research Institute, Mount Sinai Hospital, Toronto, M5G 1X5, Canada; 2 Division of General Surgery, Hepatobiliary/Pancreatic Surgical Oncology Program, Department of Surgery, University Health Network, University of Toronto, Toronto, M5S 3J3, Canada; 3 Zane Cohen Centre for Digestive Diseases Clinical Research Centre, Mount Sinai Hospital, Toronto, M5T 3L9, Canada; 4 Department of Laboratory Medicine and Pathobiology, University of Toronto, Toronto, M5S 1A1, Canada; 5 Ontario Institute for Cancer Research, Toronto, M5G 1L7, Canada; 6 Toronto General Hospital, 10EN206, 200 Elizabeth Street, Toronto, ON, M5G 2C4, Canada

**Keywords:** Hereditary cancer, Pancreas cancer, Germline variants, Genetic counseling, Carcinogenesis

## Abstract

We sequenced 11 germline exomes from five families with familial pancreatic cancer (FPC). One proband had a germline nonsense variant in *ATM* with somatic loss of the variant allele. Another proband had a nonsense variant in *PALB2* with somatic loss of the variant allele. Both variants were absent in a relative with FPC. These findings question the causal mechanisms of *ATM* and *PALB2* in these families and highlight challenges in identifying the causes of familial cancer syndromes using exome sequencing.

## Letter to the editor

*ATM* and *PALB2* variants were recently associated with familial pancreatic cancer (FPC) using exome sequencing (ES). Roberts et al. identified germline ataxia-telangiectasia (AT)-associated *ATM* variants in two FPC kindreds through ES, then in an additional 4/166 FPC probands by Sanger sequencing [1]. Jones et al. identified a frameshift variant in *PALB2* with somatic loss of heterozygosity in a patient with FPC by ES. Inactivating *PALB2* variants were found in an additional 3/96 FPC kindreds [2].

We sequenced the germline exomes of 11 affected individuals from five FPC families to search for predisposing mutations (Additional file 1: Table S1). Among genes previously associated with FPC, we identified a nonsense single nucleotide variant (SNV) in *ATM* and a nonsense SNV in *PALB2*. These SNVs were absent in 47 FPC probands, 97 in-house controls, over 6,000 control exomes, and dbSNP135 (Additional file 2).

**Figure 1:**
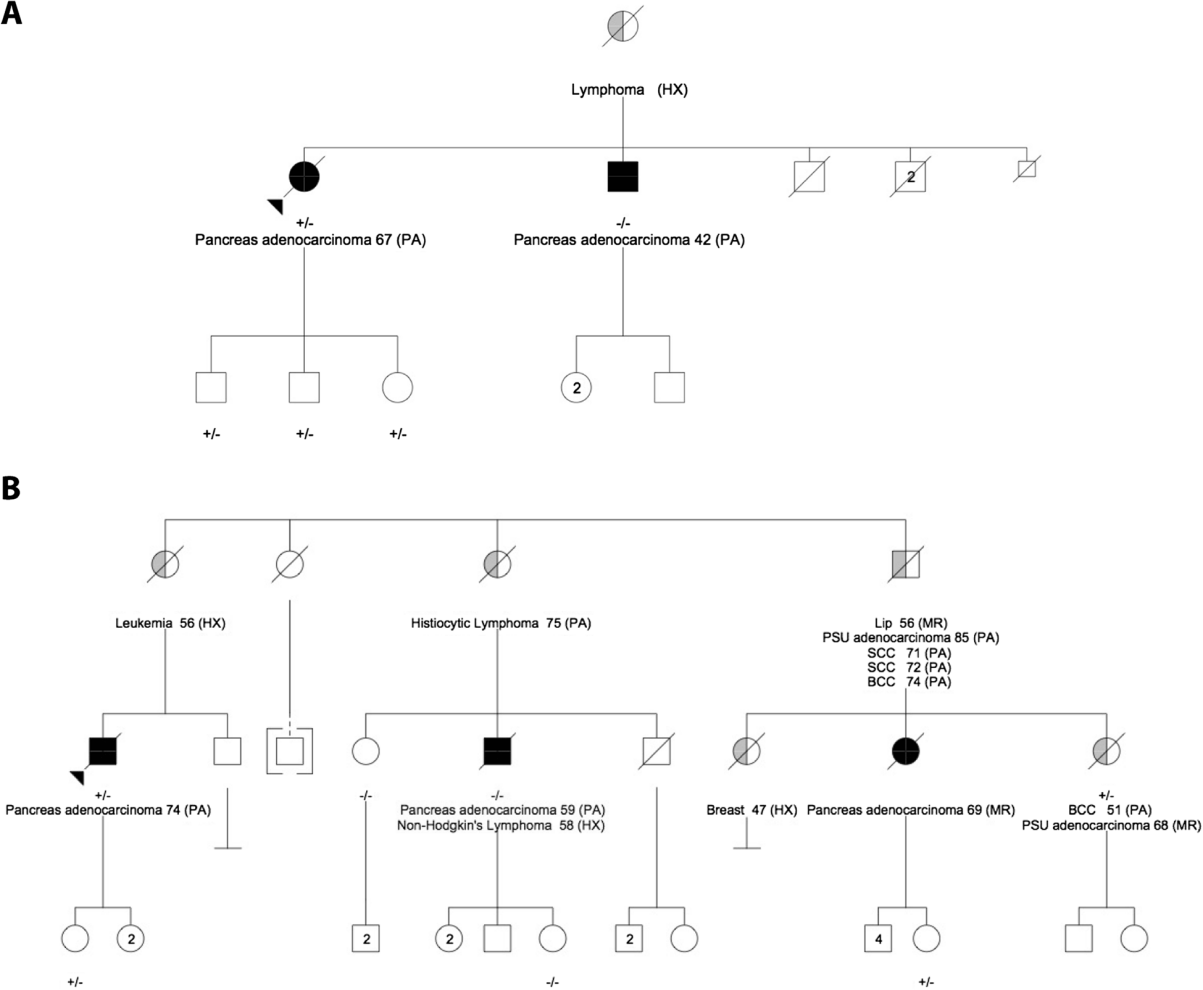
**Pedigrees of familial pancreatic cancer families, noting cancers, ages at diagnoses, and sources of diagnoses.** (**A**) Family 1 (*ATM* c.C1931A; p.S644X). (**B**) Family 2 (*PALB2* c.C3256T;p.R1086X). *HX* diagnosis from history, *PA* diagnosis from pathology reports, *PSU* primary site unknown, *SCC* squamous cell carcinoma, *BCC* basal cell carcinoma.

**Figure 2:**
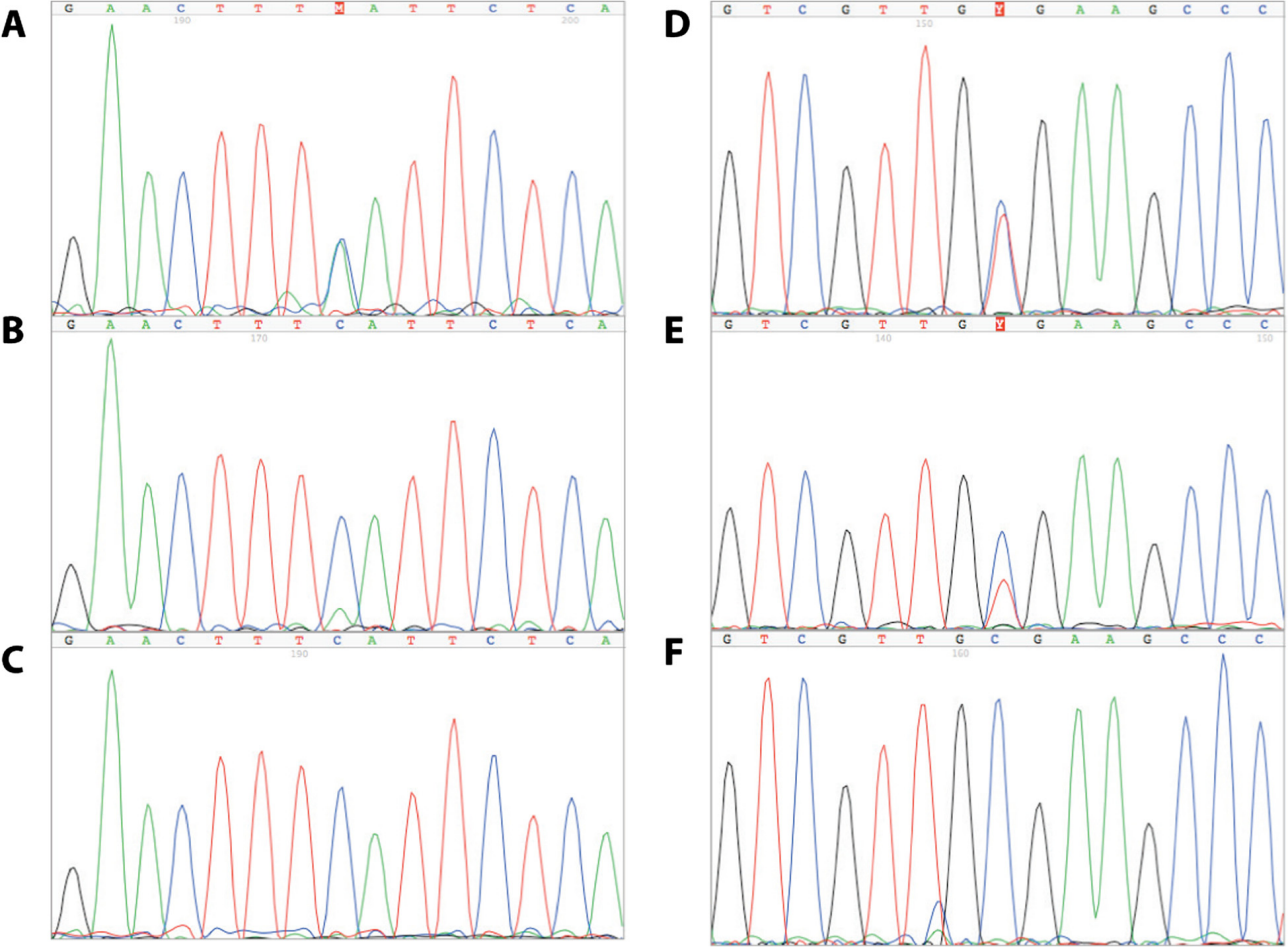
**Sequencing confirmed the exome calls and demonstrated loss of the variant alleles in matched tumors.** (**A**) *ATM* c.C1931A in the proband of family 1. (**B**) *ATM* c.C1931A in tumor of the proband of family 1, demonstrating loss of the variant allele. (**C**) Wild-type *ATM* c.C1931A in the brother with PC of the proband of family 1. (**D**) *PALB2* c.C3256T in the proband of family 2. (**E**) *PALB2* c.C3256T in the tumor from the proband of family 2, demonstrating loss of the variant allele. (**F**) Wild-type *PALB2* c.C3256T in the cousin with PC of the proband of family 2.

The proband in family 1 carried the nonsense SNV in *ATM* (c.C1931A; p.S644X), but her younger brother with FPC did not (Figure 1A; Additional file 1: Table S2). Sanger sequencing confirmed the exome calls and demonstrated loss of the variant allele in DNA from a metastasis (Figure 2A,B,C; Additional file 2). This SNV has not been previously associated with AT.

The proband in family 2 carried the nonsense SNV in *PALB2* (c.C3256T; p.R1086X), but his younger cousin with PC did not (Figures 1B and 2D,E,F; Additional file 1: Table S2, columns d,f). Sanger sequencing of the primary tumor demonstrated loss of the variant allele (Figure 2E). A third cousin with PC was an obligate carrier of the SNV (Figure 1B), as was this cousin’s sister and mother who died from metastatic adenocarcinoma from unknown primary sites (Figure 1B). This SNV was previously associated with FPC [2]*.*

Inactivating variants in *ATM* or *PALB2* are carried by approximately 0.2% of the population [3], so finding inactivating variants in two of the five kindreds supports that these genes are associated with FPC. Since the original ES studies [1,2], inactivating *PALB2* mutations were found in small proportions of FPC cohorts (for example, Tischkowitz et al. [4]), and somatic *ATM* mutations were found in 8% of sporadic pancreatic cancers [5].

However, our findings question whether *ATM* and *PALB2* predispose to FPC as ‘two hit’ tumor-suppressor genes. Both probands had somatic loss of the *variant* allele, not the wild-type allele. Moreover, both families had a relative with FPC who did not carry the mutation, with a younger age of onset than the affected carriers. These non-carrier relatives may be phenocopies, which is an important consideration for future ES studies. Alternatively, other factors may cause the familial clustering of pancreatic cancer in these kindreds.

ES is a promising technique to interrogate the genome in search of causes of complex diseases. However, ES generates thousands of candidates, and care is needed to avoid false associations. Alternative and complementary candidate gene discovery technologies such as whole-genome sequencing, copy-number analysis, and methylome analysis generate even more candidates. Since the potential for spurious findings is higher with more candidate variants, confirming associations with replication and functional studies is especially important.

Functional studies are yet to elucidate the roles of *ATM* and *PALB2* in FPC and large prospective studies assessing their associations with FPC do not exist. These considerations, combined with our findings, currently limit the interpretability and utility of clinical sequencing of *ATM* and *PALB2* in FPC.

## Abbreviations

AT: Ataxia telangiectasia; ES: Exome sequencing; FPC: Familial pancreatic cancer; PC: Pancreatic cancer; SNV: Single nucleotide variant.

## Competing interest

The authors declare that they have no competing interests.

## Authors’ contributions

RCG, WA, JDM, and SG are involved in the study concept and design, analysis and interpretation of data, and writing of the manuscript. AEB, SH, ZSK, EW, SS, TM and VP are responsible for the acquisition and analysis of data. QMT and LDS are responsible for analysis and interpretation of data. All authors read and approved the final manuscript.

## Supplementary Material

Additional file 1 **Exome sequencing results and clinical and pathological characteristics and outcomes for the individuals with pancreatic cancer in the families. Table S1**: Exome sequencing results. **Table S2**: Clinical and pathological characteristics and outcomes for the individuals with pancreatic cancer in the families with ATM c.C1931A and PALB2 c.C3256T. Click here for file

Additional file 2 **Supplement to R. C. Grant et al. exome sequencing identifies nonsegregating nonsense *ATM *and *PALB2 *variants in familial pancreatic cancer.** The file describes the supplementary methods used in the study such as patient recruitment, sample preparation, exome sequence capture and Illumina sequencing, Bioinformatics, and Sanger sequencing. Click here for file
