# Supplementary material for: Exome sequencing identifies nonsegregating nonsense ATM and PALB2 variants in familial pancreatic cancer
Source: Hum Genomics. 2013 Apr 5;7(1):11. doi: 10.1186/1479-7364-7-11 (PMC3639869; doi:10.1186/1479-7364-7-11)
Supplement: Additional file 2 — Supplement to R. C. Grant et al. exome sequencing identifies nonsegregating nonsense ATM and PALB2 variants in familial pancreatic cancer. The file describes the supplementary methods used in the study such as patient recruitment, sample preparation, exome sequence capture and Illumina sequencing, Bioinformatics, and Sanger sequencing. [file 1479-7364-7-11-S2.doc]

**Supplement to: R. C. Grant *et al.* Exome Sequencing Identifies Non-Segregating Nonsense *ATM* and *PALB2* Variants in Familial Pancreatic Cancer.**

**SUPPLEMENTARY METHODS**

**Patient recruitment**

This study was reviewed and approved by the research ethics boards at Mount Sinai Hospital, Toronto Canada (REB no: 03-0001-A) and the University Health Network (REB no: 03-0049-CE). Patients for exome sequencing were recruited through the Ontario Pancreas Cancer Study, as previously described [1], on the basis of the availability of DNA samples from multiple relatives diagnosed with pancreatic cancer (PC). Probands for follow-up sequencing were recruited based on the presence of a personal history of PC and at least one first or second-degree relative diagnosed with PC. Controls were recruited from the Familial Gastrointestinal Cancer Registry (<http://www.zanecohencentre.com/fgicr>) on the basis of an absence of personal and family history of cancer.

**Sample Preparation, Exome Sequence Capture and Illumina Sequencing**

Genomic DNA was extracted from peripheral blood lymphocytes using organic solvent isolation or column-based purification methods.

*Exome sequence capture*

The exome sequences of the patients DNA were captured over time through array hybridization with exon-targeted oligo probes using the Nimblegen HD2 sequence capture array (<http://www.nimblegen.com/products/seqcap/arrays/index.html>, Roche Nimblegen, Madison, WI), Agilent SureSelect All Exon ([www.agilient.com](http://www.agilient.com/)), or Illumina TruSeq Exome Enrichment Kit following manufacturers suggested protocols. DNA samples within any one family were treated using the same protocols.

*Whole-genome fragment libraries*

Genomic DNA was fragmented by sonication to obtain a fragment size distribution ranging from 300 base-pairs to 500 base-pairs verified via the Bioanalyzer 2100 DNA chip (Agilent). Library construction followed end repair and adaptor ligation per manufacturer protocols. The whole genome library was quantified prior to sequence capture hybridization procedure.

*Illumina Sequencing*

Post-enrichment DNA libraries of the resulting captured DNAs were sequenced on Illumina Genome Analyzer IIx or HiSeq platform with paired-end 76 to 101 base reads following the manufacturer's protocols and using standard sequencing primers. Image analyses and base callings were performed by the Genome Analyzer Pipeline with default parameters and default filtering. Two lanes per sample were used, or until we achieved coverage of at least 8 sequencing reads in 90% of the target.

**Bioinformatics**

Illumina’s latest and standard pipeline with the recommended parameters produced the raw FASTQ reads. Basic quality control (QC) metrics, including average read quality values, average base quality values, distributions of A, C, G, and T, and distributions of GC contents of all reads were performed on each lane of FASTQ reads to ensure they passed our internal QC before we proceeded to the next phase of our analysis.

Lanes that passed our QC were then aligned to the UCSC HG19 human reference genome, including random and unknown sequences, using Novoalign (www.novocraft.com) and only reads that aligned uniquely to the reference genome were kept for further analysis. Base qualities of the aligned reads were re-calibrated based on mismatches and known SNVs from dbSNP132 using the Genome Analysis Toolkit (GATK) [2]. We used GATK for local realignments around the indels, and to remove cryptic or inconsistent SNVs and indels. GATK then called SNVs and indels using the parameters recommended on the GATK wiki website (www.broadinstitute.org/gsa/wiki/index.php/The_Genome_Analysis_Toolkit). From VCF files outputted by GATK, we retained SNVs with over 7 total reads, and indels with over 11 total reads where at least 30% of which supported the alternate allele. These filters provided over 90% true positives in validation studies conducted on other samples at the Ontario Institute for Cancer Research. The remaining variants were then annotated using ANNOVAR [3] for: the predicted effect of the variant on protein sequence; and whether the variant was present in dbSNP135 [4], the March 2012 data release from the 1000 genomes project [5], or the ESP5400 data release from NHLBI GO Exome Sequencing Project [6]; and whether the mutation was in a gene previously associated with familial pancreatic cancer (FPC) (*ATM, BRCA1, BRCA2, CDKN2A, MLH1, MSH2, MSH6, PALB2, PMS2, PRSS1,* and *PRSS2, STK11, TP53*) [7]. Mutations in FPC genes considered as potentially causative were absent from the control databases and predicted to inactivate their respective protein, or were previously associated with FPC.

The kinship coefficients between all exomes were calculated using KING [8], which confirmed the expected relationships, and ruled out non-segregation due to unknown adoption or uncertain paternity.

**Sanger Sequencing**

A pathologist reviewed all tumor samples and determined the formalin-fixed paraffin embedded (FFPE) ovarian metastasis in the *ATM* c.G596A carrier approximately 45% tumor cells, and the FFPE primary tumor in the *PALB2* c.C1931A approximately 70%. Genomic DNA was extracted from peripheral blood lymphocytes and macrodissected pancreatic adenocarcinoma cells using organic solvent isolation or column-based purification methods. PCR was performed with 25ng of genomic DNA with final concentration of 1x reaction buffer (Invitrogen), 2.5mM MgCl2, 0.2mMdNTPs, 200nM of forward and reverse primers with 0.75units of Platinum Taq polymerase (Invitrogen). *PALB2* c.C1931A was amplified using the following forward and reverse primers, respectively: AGTACAGCATCACACCCACG and GGATACTTCAGAGCCTATCGGTC. *ATM* c.G596A was amplified using the following forward and reverse primers, respectively: CTCAAAGTCCGAAGAAGAGAAGC and GCTAAGTTGTCCAATTTAGAGCCC. PCR was performed on the Applied Biosystems Veriti™ 96-Well Thermal Cycler. Thermal Cycle conditions were: 95°C for 1min; 95°C for 20s, 57°C for ATM and 60°C for PALB2 for 30s, 72°C for 30s, for 35 cycles; 72°C for 5min; then hold at 10°C. Unincorporated primers and dNTPs from the PCR products were removed using Qiagen MinElute PCR Purification Kit according to the manufacturer’s protocol. The resulting DNA was prepared for sequencing using the BigDye® Terminator v3.1 Cycle Sequencing Kit, the Applied Biosystems Veriti™ 96-Well Thermal Cycler, and the Agencourt® CleanSeq purification system according to manufacturer’s instructions. Thermal cycle conditions were: 95°C for 2min, 96°C for 10s, 50°C for 5s, for 25 cycles; 60°C for 4min; hold at 10°C. Sequencing was performed on the ABI 3130xl or 3730xl genetic Analyzer using the standard FastSeq50_POP7_1 program. Chromatograms were analyzed for variants discordant with the reference genome hg19 using 4peaks ([www.mekentosj.com/science/4peaks](http://www.mekentosj.com/science/4peaks)) assembly, alignment, and analysis software. Nonsynonymous variants with less than 5% allele frequency in the 1000 genomes project were re-sequenced and confirmed in the reverse direction (**Figure S1**).

**References**

**1.** Borgida AE *et al*., *Can J Surg.* 2011; 54(1): 54-60.

**2.** DePristo M *et al.,* *Nature Genetics* 2011; 43(5): 491-498.

**3.** Wang K *et al., Nucleic Acids Research* 2010**;** 38:e164.

**4.** Database of Single Nucleotide Polymorphisms (dbSNP). Bethesda (MD): National Center for Biotechnology Information, National Library of Medicine. (dbSNP Build ID: 135). Available from: <http://www.ncbi.nlm.nih.gov/SNP/>.

**5.** Mills RE *et al., Nature* 2010; 470(7332): 59-65.

**6.** Exome Variant Server, NHLBI Exome Sequencing Project (ESP), Seattle, WA. (<http://evs.gs.washington.edu/EVS/>): accessed April 10, 2012.

**7.** Roberts NJ *et al.,* *Cancer Discovery* 2011; 2(1): 41-46.

**8.** Manichaikul A *et al*., *Bioinformatics* 2010; 26(22): 2867-73.

| **A.**  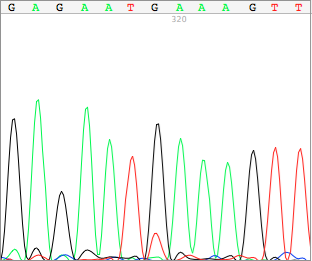 | **B.**  **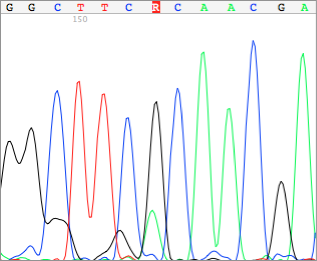** |
| --- | --- |

**Figure S1:** Chromatograms from the tumors of the carriers of *ATM* c.C1931A (**A**) and *PALB2* c.C3256T (**B).** demonstrating reduced peak for the variant allele compared to the germline chromatograms in Figure 1.
